# Hydrothermal Synthesis of γ‑Al_2_O_3_ Nanofluids for Enhanced Oil Recovery: Characterization and Coreflooding Evaluation

**DOI:** 10.1021/acsomega.5c05087

**Published:** 2025-09-03

**Authors:** Camila Louyse Oliveira da Rocha, Ana Leticia Santos, Paulino Vasco Mariano Muguirrima, Gregory Vinicius Bezerra de Oliveira, Armando Monte Mendes, Marcos Allyson Felipe Rodrigues, Antonio Eduardo Martinelli

**Affiliations:** † 28123 Federal University of Rio Grande do Norte, Av. Senador Salgado Filho No. 3000, Natal 59078-900, Rio Grande do Norte, Brazil; ‡ Faculty of Science and Technology, Zambezi University, Beira 2100, Mozambique

## Abstract

Nanoparticles are increasingly used in enhanced oil recovery (EOR) due to their resistance to harsh conditions and effective oil dispersion. Al_2_O_3_ nanoparticles, in particular, can reduce interfacial tension (IFT) and alter rock wettability, improving oil mobility. This study synthesized γ-Al_2_O_3_ nanoparticles via a low-temperature, short-duration hydrothermal method, prepared nanofluids, and evaluated their performance in medium-viscosity oil recovery. Nanoparticles were characterized by X-ray diffraction (XRD), field emission scanning electron microscopy (FESEM), Fourier transform infrared spectroscopy (FTIR), and BET surface area analysis. Nanofluids were prepared in saline solution at different concentrations and assessed for colloidal stability by UV–vis spectroscopy and sedimentation tests, along with rheological properties and interactions with rock and oil. EOR tests used Buff Berea sandstone plugs. XRD confirmed γ-Al_2_O_3_ formation without secondary phases. FTIR, FESEM, and BET analyses showed characteristic alumina bonds, uniform nanoplate morphology, and high specific surface area. The 0.01 wt % nanofluid remained stable for 2 h, while higher concentrations resulted in sedimentation and aggregation. Nanofluids exhibited Newtonian behavior with viscosity similar to water. IFT decreased as nanoparticle concentration increased; however, efficiency dropped at 0.10 wt % due to agglomeration. Wettability tests after 24 h indicated that 0.01 wt % reduced the contact angle to 64°, whereas 0.05 and 0.10 wt % fully reversed wettability (0°). In oil recovery, 0.10 wt % γ-Al_2_O_3_ nanofluid achieved the highest recovery (>29%) versus brine (26.31%). Appropriate nanoparticle concentration is essential to optimize recovery efficiency.

## Graphical abstract

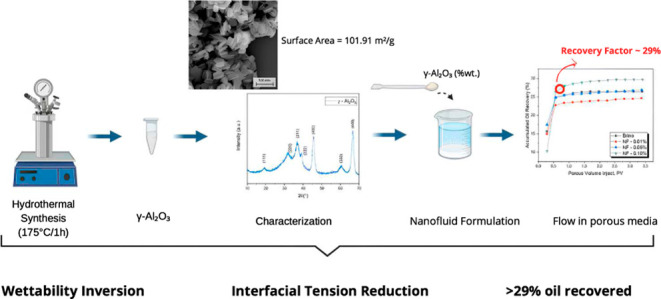

## 1 Introduction

Enhanced oil recovery (EOR) techniques can be classified into thermal, miscible, microbial, or chemical methods.^1^ These approaches promote interactions between the injected fluid and the trapped oil or modify the reservoir rock properties, resulting in an increased recovery factor.^1^ Among these strategies, chemical recovery stands out due to the use of surfactants, polymers, alkalis, and nanoparticles, which directly influence oil–rock–fluid interactions, optimizing the extraction of residual oil.^2–4^

Nanomaterials have been prominent in chemical oil recovery due to their resistance to adverse reservoir conditions, ability to penetrate rock pores and efficiency in dispersing oil, preventing its deposition.^5–7^ In the reservoir, these materials can act by different mechanisms, such as disjoining pressure rock wettability inversion,^8–13^ and reduction of capillary force improving oil mobility.^14,15^

The study conducted by Lu et al. (2016)^7^ indicates that nanoparticles can also adsorb asphaltenes on their surface, contributing to improved recovery efficiency. Asphaltenes are heavy petroleum compounds that, when deposited in rock pores, alter wettability and reduce recovery efficiency.^16–18^ Their concentration and molecular size vary according to the API gravity of the oil, being more abundant in heavy oils.^19,20^ In this context, nanoparticles have shown potential to mitigate these negative effects by promoting asphaltene dispersion and reducing interfacial tension (IFT), thereby enhancing oil flow.^7^

Nanoparticle synthesis can be performed using various methods, such as sol–gel,^21^ coprecipitation,^22^ spray pyrolysis,^23^ microwave-assisted solvothermal,^24^ and hydrothermal synthesis.^25^ Among these, hydrothermal synthesis stands out for its precise control over parameters such as time, temperature, and pH, enabling the production of nanostructured materials with well-defined morphology and high surface area, which are ideal for EOR applications. Additionally, it is an environmentally friendly approach, as it uses water as the reaction medium, avoiding toxic solvents.^25,26^

This technique is commonly used in the production of EOR materials such as SiO_2_,^27^ TiO_2_,^28^ ZnO^29^ and Al_2_O_3_.^30^ The latter is particularly relevant due to its ability to reduce IFT, reverse rock wettability and resist severe well condition.^30^ Authors such as Minaei et al. 2020 ^25^ synthesized Al_2_O_3_ nanoparticles by hydrothermal synthesis, using precursors such as aluminum nitrate and bases such as NaOH or KOH. The mixture was placed in a hydrothermal reactor at 200 °C for 24 h. In another study, Wang et al. 2021 ^31^ used aluminum sulfate, CTAB and urea as reagents, subjecting the solution to hydrothermal synthesis at 165 °C for 24 h, also obtaining Al_2_O_3_ nanoparticles with controlled properties.

γ-Al_2_O_3_ also stands out for its high thermal and chemical stability and large specific surface area. Nanoplates of γ-Al_2_O_3_, in particular, have demonstrated superior performance in viscosity reduction and wettability alteration when compared to spherical or rod-shaped particles.^32^ Moreover, aluminum precursors are low-cost and widely available, contributing to the economic feasibility of large-scale applications.

Nanofluids are colloidal dispersions of nanoparticles in base fluids, such as water or brine. Their application in EOR has been extensively investigated due to their effectiveness in improving oil extraction by different mechanisms.^10^ For instance, nanofluids containing SiO_2_ and TiO_2_ form thin films that reduce oil adhesion to rock surfaces,^33^ whereas ZnO, Al_2_O_3_, and ZrO_2_ exhibit excellent performance in wettability reversal, even at low concentrations.^34,35^ Fe_2_O_3_ nanofluids, in turn, are effective in decreasing capillary forces, facilitating the mobilization of trapped oil, thereby enhancing recovery efficiency.^15,36^

This study aims to synthesize high surface area γ-Al_2_O_3_ nanoparticles by the hydrothermal method under low temperature and time and evaluate its performance in enhanced oil recovery. Nanofluids were formulated to evaluate their performance in medium viscosity oils (°API ≈ 22). The investigation included analyses of the interactions between the nanofluids and the oil, as well as their influence on the interfacial tension and wettability of the rock. Tests on Buff Berea sandstone plugs were conducted to evaluate the recovery efficiency under controlled reservoir conditions.

## 2 Materials and Methods

### 2.1 Nanoparticle Synthesis

The synthesis conducted in this study is based on the formation of the boehmite phase (AlO­(OH)),^37^ which can be thermally treated to obtain γ-Al_2_O_3_.^38^ Aluminum nitrate nonahydrate (Al­(NO_3_)_3_·9H_2_O, Sigma-Aldrich, ≥98%) was used as the aluminum precursor, urea (CH_4_N_2_O, Dinâmica, 99%) as a controlled hydrolysis agent, and distilled water as solvent. A molar ratio of approximately 1.61:1 (urea/aluminum nitrate) was employed. Initially, 0.00445 mol of Al­(NO_3_)_3_·9H_2_O and 0.00716 mol of urea were dissolved in 100 mL of distilled water under magnetic stirring until complete dissolution. The solution was then transferred to a 100 mL PTFE-lined stainless steel autoclave and kept at 175 °C for 1 h under autogenous pressure and constant stirring. After cooling, the product was centrifuged at 5500 rpm for 10 min and washed twice with water. The recovered material was oven-dried at 80 °C for 10 h and subsequently calcined at 550 °C for 6 h, with a heating rate of 10 °C/min.

The synthesis parameters, temperature of 175 °C and reaction time of 1 h, were selected based on initial reports in the literature, which typically employ temperatures ranging from 165 to 200 °C and durations up to 24 h for hydrothermal synthesis of Al_2_O_3_ nanoparticles.^25,31^ Preliminary tests in this study confirmed that these milder conditions were sufficient to promote the formation of the boehmite phase, which was successfully converted to γ-Al_2_O_3_ upon calcination, as confirmed by XRD. The use of lower temperature and shorter duration was intended to reduce energy consumption and processing time while maintaining the desired crystallinity and morphology, thereby enhancing the method’s efficiency. The synthesis steps are shown in Figure 1.

**Figure 1:**
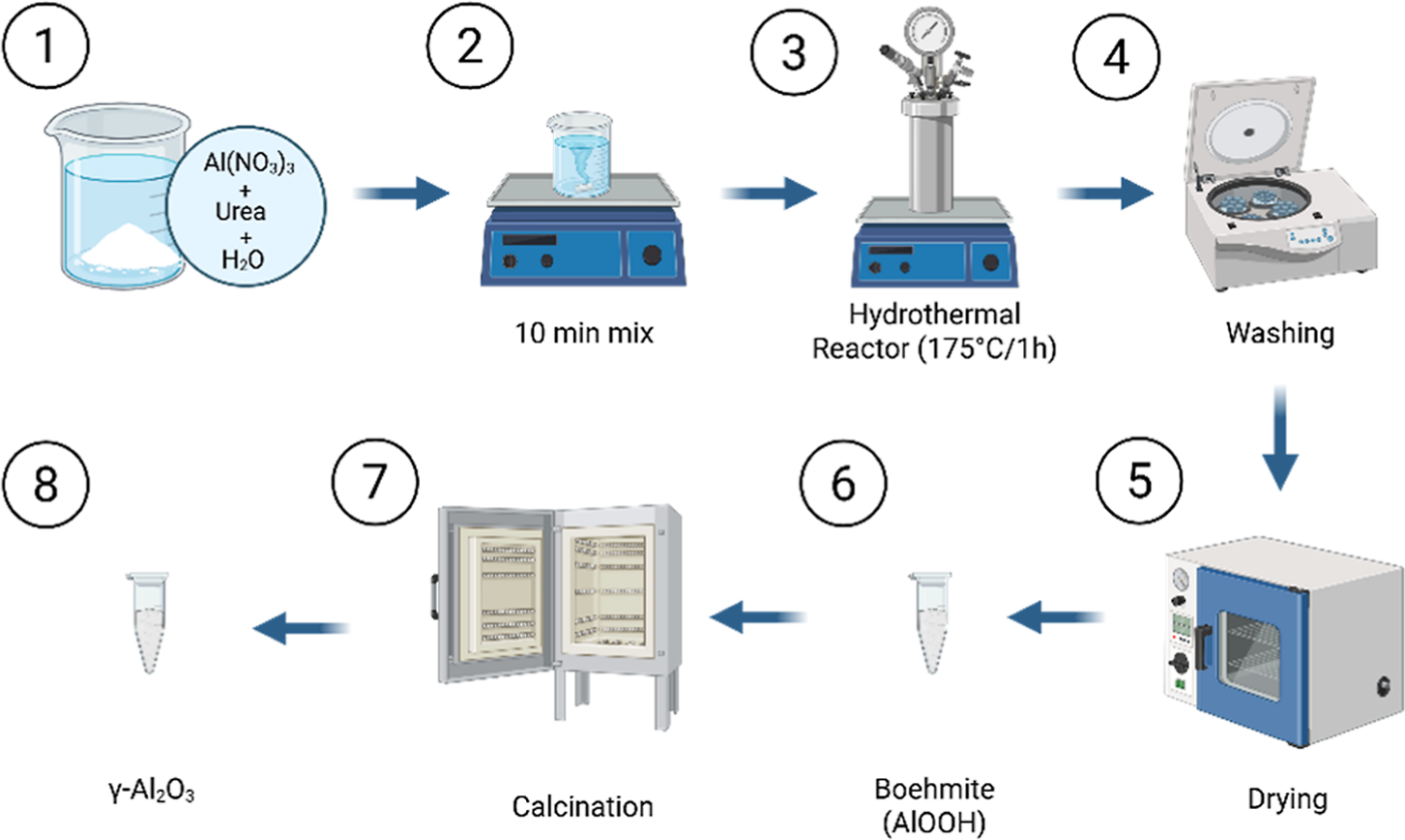
Schematic representation of the γ-Al_2_O_3_ nanoparticle synthesis process: (1) dissolution of precursors in distilled water, (2) magnetic stirring, (3) hydrothermal treatment, (4) washing and separation, (5) drying, (6) formation of boehmite, (7) calcination, and (8) formation of γ-Al_2_O_3_ nanoparticles. Created in BioRender.

### 2.2 Characterization of Nanoparticles

The synthesized materials were characterized by X-ray diffraction (XRD) on a Shimadzu XRD-7000 diffractometer, using Cu Kα radiation and set to 40 kV for the angular scanning range of 10° ≤ 2θ ≤ 70°, both before and after calcination, to evaluate its crystallographic characteristics. Rietveld refinement was applied to the XRD data to obtain detailed information about the crystal structure of γ-Al_2_O_3_, using the Materials Analysis Using Diffraction (MAUD) software. The reliability of the refinement was evaluated by the residue weighting index (*R*_wp_), the expected weighted profile factor (*R*_exp_), the Bragg factor (*R*_B_) and quality of fit (χ^2^). Low values for *R*_wp_, *R*_exp_ and *R*_B_, together with a χ^2^ close to 1, indicate a good fit between the observed and calculated data, confirming the accuracy and reliability of the refinement. Field emission scanning electron microscopy (FEG-SEM) was also used, with the ZEISS Auriga 40 model, to evaluate the dimensional and morphological characteristics of the postsynthesized samples. Additionally, the characterization of the chemical bonds present in the samples was performed by Fourier transform infrared spectroscopy (FTIR), using the BRUKER Vertex 70 model, coupled to an attenuated total reflection (ATR) device with a diamond prism. The specific surface area of the samples was determined by the Brunauer–Emmett–Teller (BET) method, using the BELSORPminiII model from BEL Japan, based on the physical adsorption data of N_2_.

### 2.3 Preparation and Characterization of γ-Al_2_O_3_ Nanofluids (NFs)

Initially, a saline solution containing 2% KCl (Synth) was prepared, as this concentration is commonly adopted as a standard brine in enhanced oil recovery (EOR) experiments to prevent clay swelling in sandstone formations.^39^ Then, Al_2_O_3_ nanoparticles were incorporated into the solution at concentrations of 0.01%, 0.05%, and 0.10% by weight. To ensure accurate and reproducible formulations, the nanoparticles were weighed using an analytical balance and added to the saline solution under controlled conditions. Both solutions underwent a nanoparticle dispersion process in an ultrasonic bath (USC-1800, Ultronic, 25 kHz, 160 W) for 15 min to promote homogeneous distribution and minimize agglomeration at room temperature. After sonication, each nanofluid was visually inspected to check for signs of sedimentation or phase separation. Rheological measurements were performed immediately after preparation using a Thermo Fisher Scientific Haake Mars rheometer to analyze the rheological behavior and viscosity of the nanofluids, maintained at 25 °C with a shear rate ranging from 0.1 to 1000 s^–1^. From the collected data, shear rate vs shear stress and shear rate vs viscosity curves were generated.

To further investigate the dispersion behavior and short-term stability of the nanofluids, UV–vis spectroscopy analyses were performed. Optical absorption spectra of the samples (0.01, 0.05, and 0.10 wt % Al_2_O_3_) were recorded using an IL-592 UV–vis spectrophotometer equipped with tungsten and D2 lamps. Measurements were performed over a wavelength range of 200–900 nm, with a resolution of 1 nm, using quartz cuvettes. The nanofluid samples were analyzed at 30 min intervals for a total duration of 2 h. Data acquisition was performed using MetaSpec software.

To complement the UV–vis spectroscopy results and further evaluate the short-term colloidal stability of the nanofluids, a sedimentation test was conducted through direct visual inspection. Nanofluid samples containing 0.01, 0.05, and 0.10 wt % of Al_2_O_3_ were stored in transparent glass vials at room temperature and photographed at defined time intervals (0 h, 10 min, 20 min, 30 min, 1 h, 1 h 30 min and 2 h). This procedure allowed for qualitative assessment of sedimentation behavior and nanoparticle dispersion over time, providing visual evidence of stability trends observed in the UV–vis analysis.

### 2.4 Fluid–Fluid and Rock–Fluid Interaction Tests

To evaluate the interaction of the nanofluid with the oil sample (*d* = 0.918 g/mL; μ = 78.2 cP; BSW = 1.2%) and the rock (Berea sandstone) used in the enhanced recovery tests, interfacial tension and wettability tests were performed. The interfacial tension test was performed on a Krüss force tensiometer K20 using the Du Noüy ring method at 25 °C. Each measurement was performed in duplicate (*n* = 2), and the results are presented as mean ± standard deviation. Table 1 lists the fluids used in the IFT experiments.

**Table 1:** Fluids Used in the IFT Test

fluid number	composition
1	brine–oil
2	NF 0.01 wt % Al_2_O_3_–oil
3	NF 0.05 wt % Al_2_O_3_–oil
4	NF 0.10 wt % Al_2_O_3_–oil

To evaluate the influence of nanofluids on the wettability of Berea sandstone, the contact angles between a 2% KCl brine solution and the surface of oil-saturated rock pellets treated with nanofluids were measured. Sample preparation followed the methodology of de Oliveira et al. (2023).^39^ The Berea sandstone plugs were sectioned into ∼0.5 cm disks, dried at 80 °C for 24 h and subsequently immersed in the oil sample for 48 h at 60 °C. After this step, the samples were washed with toluene and *n*-heptane, dried at room temperature for 24 h, and subsequently immersed in the nanofluids for two different treatment durations: 30 min and 24 h. Subsequently, the samples were analyzed using the appropriate equipment. The contact angle was determined with a Krüss goniometer (DSA100), applying a 5 μL drop of 2% KCl on the surface of the tablet. Each measurement was conducted in duplicate (*n* = 2), and values are reported as mean ± standard deviation.

### 2.5 Petrophysics of the Berea Buff Sandstones

Enhanced recovery tests were performed using 1.5 in. diameter, 3 in. long Buff Berea sandstone (HICO) plugs, which are composed predominantly of quartz and are naturally water-wet. These plugs were characterized in terms of petrophysical properties, such as porosity and permeability. Porosity was measured using the HEP-E Educational Helium porosimeter from Vinci Technologies, applying Boyle’s law. Permeability, in turn, was determined using the GPE 100 Educational steady-state gas permeameter, also from Vinci Technologies, which assesses the permeability of the rock to N_2_ gas in steady-state conditions. This analysis follows the concept of Darcy’s Law, with subsequent correction for the Klinkenberg effect.

### 2.6 Flow Tests in Porous Media

The methodology used was based on the work developed by de Oliveira et al. (2023).^39^ Initially, the plugs were saturated with 2% KCl brine in a Vinci Technologies vacuum saturator under 2000 psi for 2 h. After saturation, the volume of brine that invaded the pores of the plugs was determined by means of a mass balance, comparing the dry mass and the wet mass of the rock, considering the density of the solution. The flow tests were performed on the CFS-700 system from Vinci Technologies. The equipment diagram is presented in the work of de Oliveira et al. (2023).^39^ The plugs were saturated with oil by injecting two pore volumes (PV) at 0.5 mL/min and 1 mL/min, determining the initial oil volume (*V*_oi_) and initial oil saturation (*S*_oi_). In the enhanced recovery tests, separate flow tests were performed, each conducted independently at a flow rate of 0.25 mL/min. The experiments included: (i) a test with exclusive brine injection, (ii) individual tests with injection of nanofluids containing 0.01%, 0.05% and 0.10% Al_2_O_3_ and (iii) a test combining brine injection followed by the nanofluid that showed the best performance in the previous tests. Effluent samples of approximately 5 mL were collected, and the total volume of oil recovered (*V*_rec_) was verified. The residual oil saturation (*S*_or_) and the recovery factor were calculated based on *V*_oi_, *V*_rec_ and pore volume, according to eqs 1 and 2.

1 $$S_{\text{or}}=\frac{V_{oi}-V_{rec}}{PV}$$

2 $$\%\;FR=\left(\frac{V_{rec}}{V_{oi}}\right)\times 100$$

For each flow test, the produced fluids were collected at the core holder outlet in Falcon tubes. After collection, the tubes were centrifuged at 2500 rpm for 10 min to facilitate the separation between the aqueous and oily phases. Based on these data, a mass balance was performed to determine the final volumes of oil and water collected and to calculate the recovery parameters mentioned above. Table 2 presents a summary of the combinations and configurations of the core injection tests performed herein, as well as the parameters evaluated in each experiment.

**Table 2:** Flow Tests Performed

coreflooding test number	injected fluid	evaluated parameter
1	brine	fluid effect
2	NF 0.01 wt % Al_2_O_3_	effect of nanoparticle concentration on recovery
3	NF 0.05 wt % Al_2_O_3_	effect of nanoparticle concentration on recovery
4	NF 0.10 wt % Al_2_O_3_	effect of nanoparticle concentration on recovery
5	brine + NF 0.10 wt % Al_2_O_3_	effect of nanofluid injection after brine injection

## 3 Results and Discussion

### 3.1 Crystal Structure Evaluation

X-ray diffraction analyses revealed the synthesis of the boehmite phase (Figure 2a), identified according to the crystallographic standard ICSD 15696. The average crystallite size was approximately 11 nm according to the Scherrer method. After calcination of the boehmite powder, a new XRD analysis was conducted, illustrated in Figure 2b, to confirm the phase transformation to γ-Al_2_O_3_ (COD 2015539).

**Figure 2:**
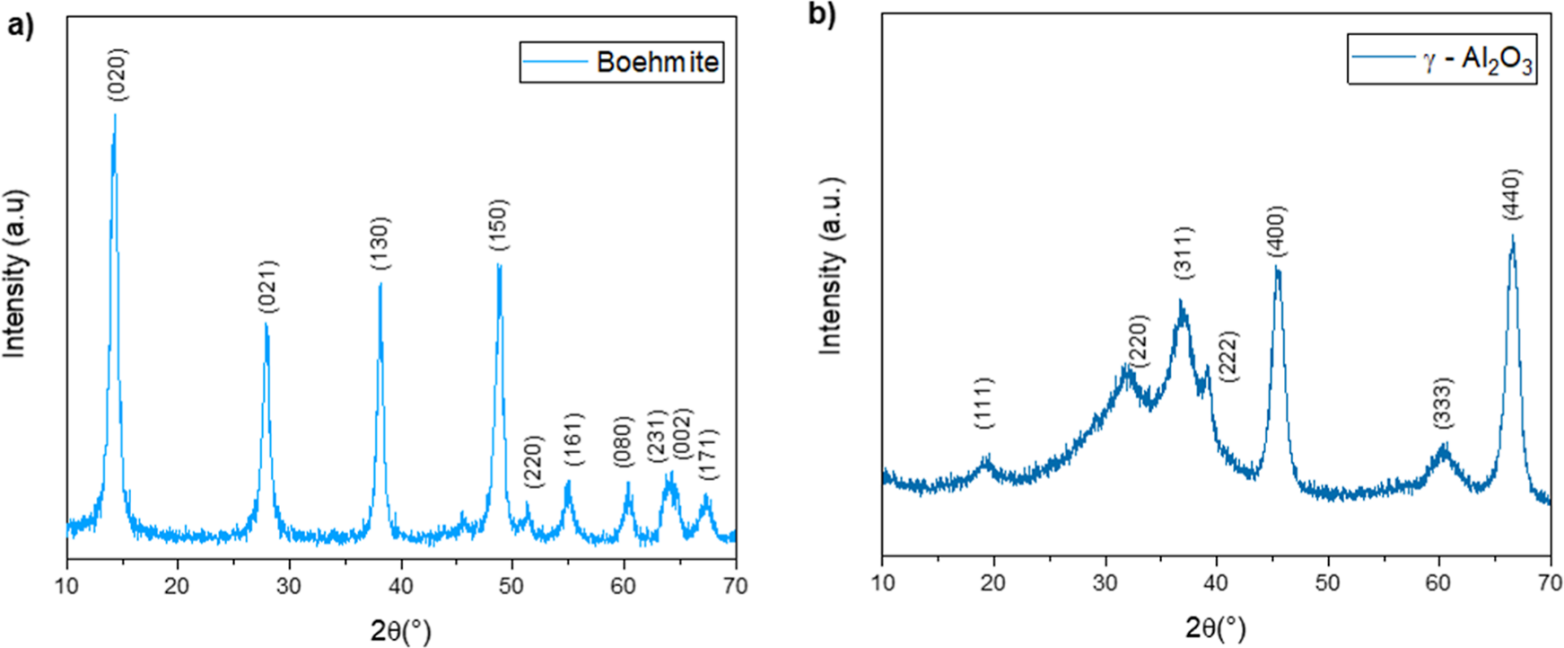
XRD of (a) boehmite and (b) γ-Al_2_O_3_ powders.

The analysis revealed the presence of the cubic phase γ-Al_2_O_3_ without secondary phases. The broad diffraction peaks indicate a small crystallite size of approximately 6 nm, which is advantageous for applications that require high surface area, such as EOR. This characteristic directly impacts key properties like thermal stability, catalytic activity, and mechanical strength, which are crucial for maintaining nanoparticle integrity under reservoir conditions. These properties are influenced by the *Fd*3̅*m* crystal structure of γ-Al_2_O_3_, which resembles a deformed spinel due to similarities in atomic packing and the arrangement of cationic vacancies. Both structures exhibit a face-centered cubic (FCC) oxygen lattice; however, the cationic positions in γ-Al_2_O_3_ are disordered, generating local lattice distortions. These distortions, represented in the structural model generated by the VESTA software (Figure 3), play a significant role in enhancing the material’s chemical reactivity and stability under harsh operating conditions.^40^

**Figure 3:**
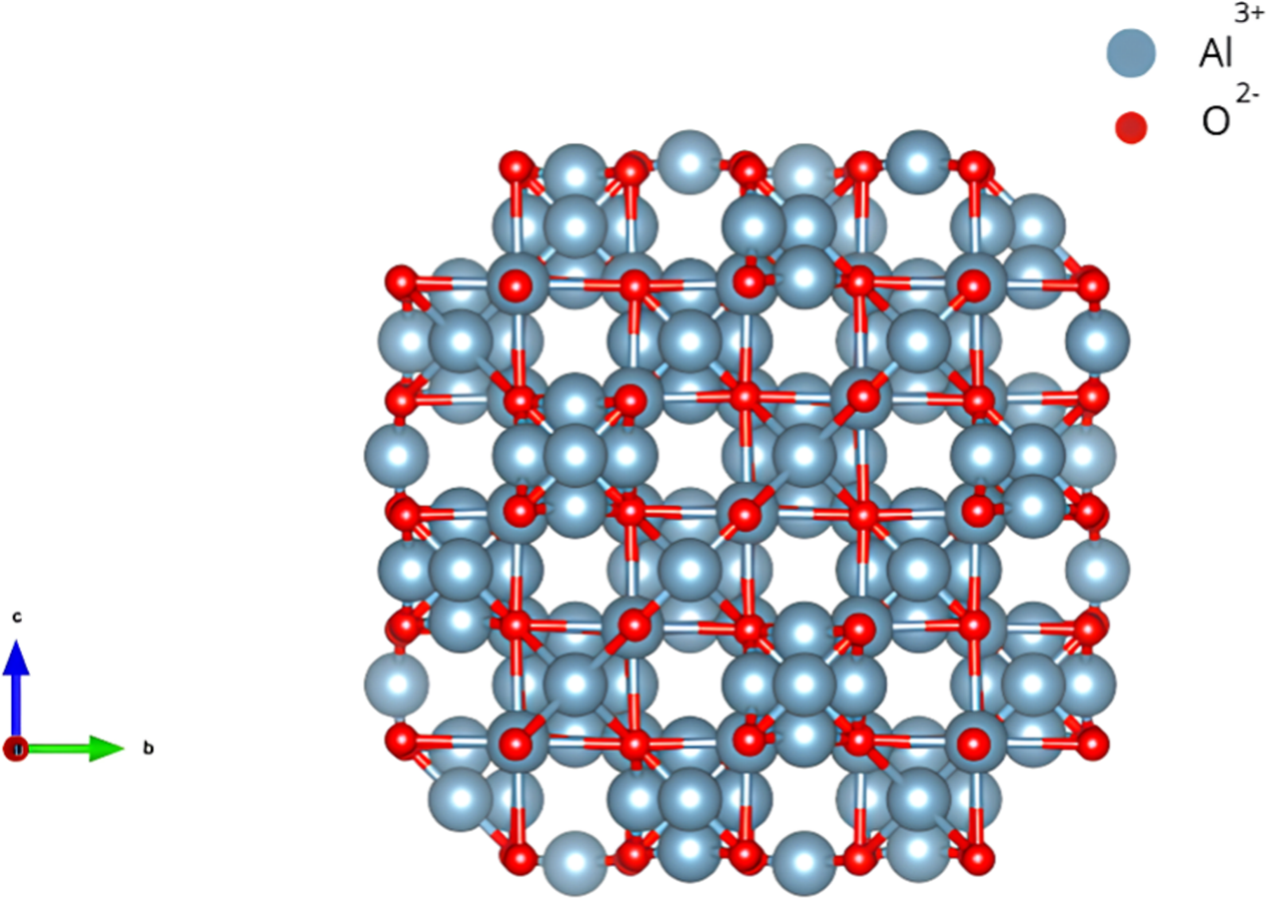
Atomic structure of γ-Al_2_O_3_ with a cubic *Fd*3̅*m* lattice. Blue spheres represent Al^3+^ cations and red spheres represent O^2–^ anions.

For more detailed information on the structure of the synthesized material, Rietveld refinement was conducted and the data obtained can be seen in Figure 4 and Table 3. The refinement by the Rietveld method confirmed the quality of the fit of the structural parameters, with values of *R*_wp_ (10.90%) and χ^2^ (1.57) indicating good convergence between the experimental data and the theoretical model. These results demonstrate the validity of the *Fd*3̅*m* structure of γ-Al_2_O_3_ with a lattice parameter of approximately 7.92 Å.

**Figure 4:**
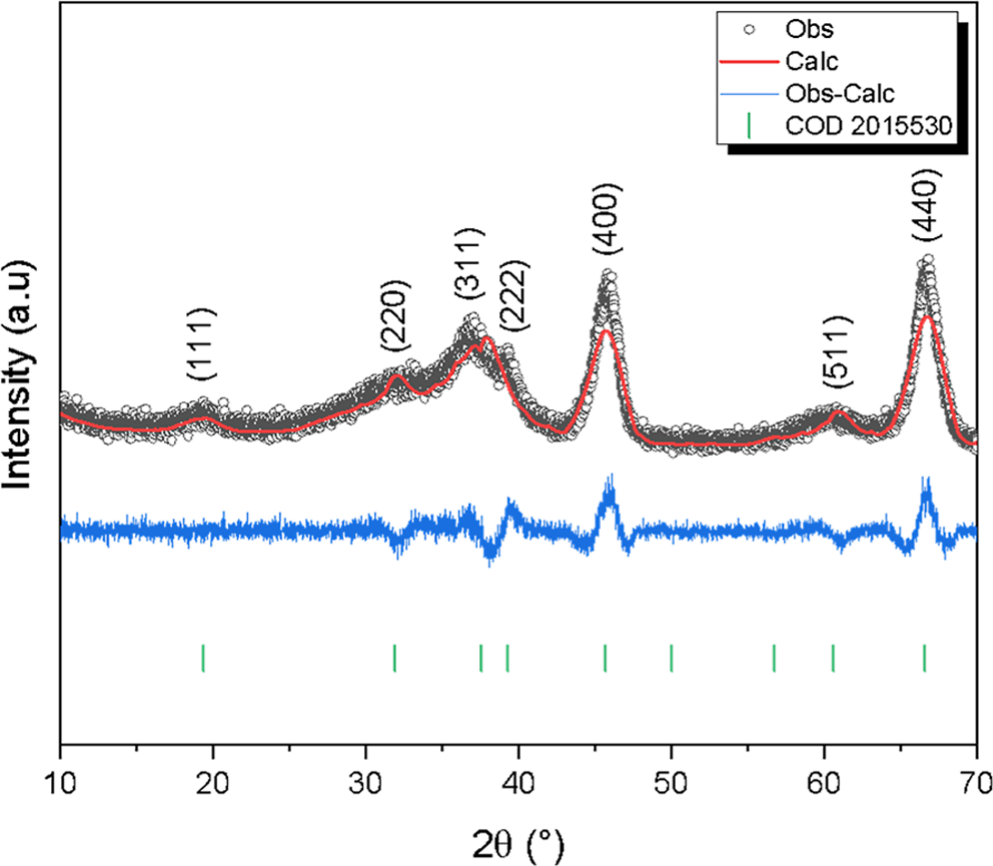
XRD refinement of γ-Al_2_O_3_.

**Table 3:** Parameters Obtained from Rietveld Refinement of γ-Al_2_O_3_

parameters	γ-Al_2_O_3_
*a* (Å)	7.92
volume (*a* ^3^)	496.79
*R*_B_ (%)	8.92
*R*_wp_ (%)	10.90
*R*_exp_ (%)	6.90
χ^2^	1.57
crystallite size (nm)	6.06
microdeformation (10^–4^)	0.00243

### 3.2 Morphology and Particle Size Distribution

The micrograph shown in Figure 5 illustrates the morphology of boehmite powder. The particles exhibit a uniform plate-like morphology with low agglomeration, which promotes homogeneous material distribution.

**Figure 5:**
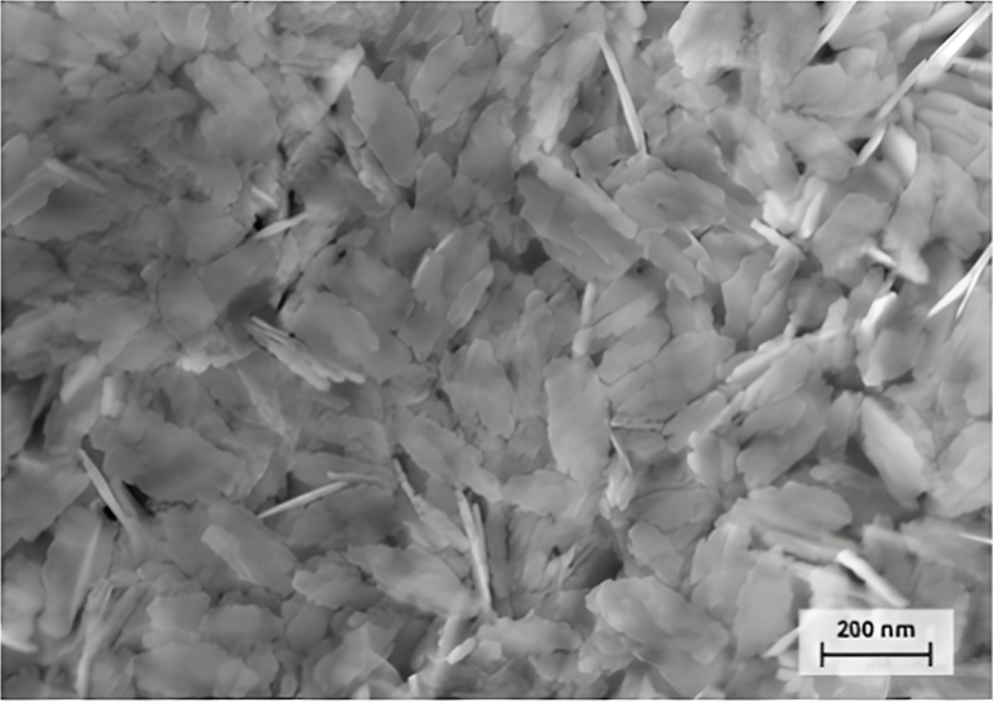
Micrograph of boehmite.

The observed nanoplate-like morphology of boehmite may be related to a lateral aggregation mechanism during crystal growth, as suggested by the presence of irregular edges at the particle boundaries. Such features are often associated with anisotropic growth from small, nucleated domains, resulting in thin, plate-shaped particles.

The micrograph shown in Figure 6a illustrates the morphology of the γ-Al_2_O_3_ powder. It can be observed that, even after calcination, the uniform nanoplate morphology was preserved, with dimensions of approximately 100 nm in length and 30 nm in thickness. These values were confirmed through particle size distribution analysis, in which the dimensions of the nanoplates were measured using ImageJ software. The collected data were then processed and histograms plotted using Origin software, as shown in Figure 6b,c. Additionally, the material exhibited a specific surface area of 101.91 m^2^/g, as determined by the BET method.

**Figure 6:**
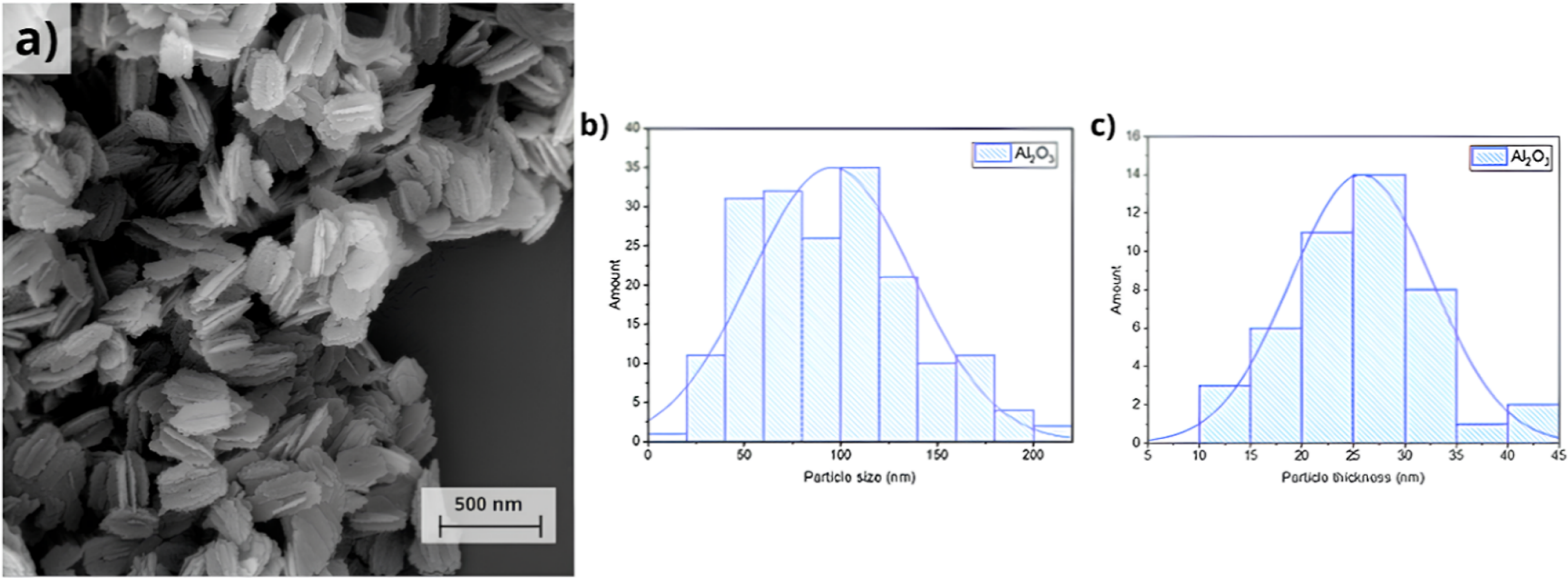
(a) Micrograph of γ-Al_2_O_3_ (b) particle size distribution of γ-Al_2_O_3_ with respect to length (c) and thickness.

The crystallite size estimated for γ-Al_2_O_3_ by the Scherrer equation was approximately 6 nm, suggesting that each nanoplate consists of multiple nanoscale building blocks. This indicates that the particles are polycrystalline,^41^ which helps explain the apparent discrepancy between the crystallite size obtained by X-ray diffraction (XRD) and the overall particle dimensions observed in FESEM images.

The specific surface area (SSA) of the sample was determined by BET analysis, resulting in a high value of 101.91 m^2^/g. This elevated SSA is strongly associated with the morphology of the nanoparticles, which predominantly exhibit a nanoplate-like structure with an average thickness of approximately 30 nm, as confirmed by FESEM observations. To validate this correlation, the theoretical SSA was estimated based on a simplified geometric model that treats the nanoparticles as thin, planar plates with surface area dominated by their two largest faces and negligible edge contributions. This approach is commonly used in the literature to approximate SSA from morphological features when particles deviate from spherical geometry.^42,43^ Using the density of γ-Al_2_O_3_ (∼3.65 g/cm^3^) and the average thickness (∼30 nm), the theoretical SSA (eq 3) is approximated by

3 $${SSA}_{theoretical}=\frac{total\;surface\;area}{mass}=\frac{2\times A_{face}}{V\times \rho }=\frac{2\times A_{face}}{A_{face}\times t\times \rho }=\frac{2}{\rho \times t}\approx 182\;m^{2}/g$$

Although this theoretical value is higher than the experimental result, such deviation is expected due to factors like nanoparticle agglomeration, structural irregularities, and limited accessibility to internal or occluded surfaces. Still, the agreement in order of magnitude supports the conclusion that the high SSA is consistent with the nanoplate morphology observed.

It is important to highlight that the obtained surface area of 101.91 m^2^/g is within or above the range reported in the literature for γ-Al_2_O_3_ nanoparticles used in EOR applications. For example, Izadi and Nasernejad (2022)^7^ reported a γ-Al_2_O_3_ with a surface area of 90.25 m^2^/g that demonstrated good performance in altering wettability.^70^ This confirms that the SSA achieved in this work is suitable for EOR applications, since a high surface area facilitates surface-mediated mechanisms such as interfacial tension reduction, asphaltene adsorption, and wettability modification.^7^

A high degree of agglomeration was observed in the micrographs, as indicated by the significant difference between the crystallite size estimated by XRD and the particle dimensions seen in FESEM. This agglomeration reduces the availability of active surface area for interactions in EOR processes, which are largely surface-dependent. However, the calcination conditions used were sufficient to induce the phase transformation to γ-Al_2_O_3_ while avoiding excessive sintering. This helped to preserve the nanoscale characteristics necessary for EOR applications, even though some particle aggregation was unavoidable.

Studies indicate that the plate-shaped morphology of γ-Al_2_O_3_ is particularly effective in reducing oil viscosity, especially heavier oils, in addition to modifying rock wettability, favoring increased oil recovery.^32,44^ According to Manesh et al. (2017),^32^ γ-Al_2_O_3_ nanoplates at a concentration of 0.30 wt % were able to reduce oil viscosity, outperforming other morphologies such as spherical and rod-shaped. This superior performance is attributed to the larger surface area and heat transfer capacity of the nanoplates, which promote better oil flow through the pores of the reservoir rocks.

### 3.3 Chemical Analysis of Particles

From the analysis of Figure 7, it is possible to observe the formation of the main bonds in alumina: O–H (3460 cm^–1^ and 1637 cm^–1^), corresponding to the elongation and curvature of the hydroxyl groups (OH), respectively. The presence of OH groups indicates the interaction of alumina with the external environment. Such interaction tends to increase as its specific surface area increases.^45^ Furthermore, the hydroxyl groups present on the alumina surface can facilitate chemical reactions with the rock surface or with other species in the environment, such as asphaltenes found in petroleum, which can be adsorbed on the alumina surface, thus contributing to improved recovery.^7,30^ Additionally, the Al–O bond was identified at 511 cm^–1^, confirming the presence of aluminum atoms bonded to oxygen atoms in the alumina crystal lattice.

**Figure 7:**
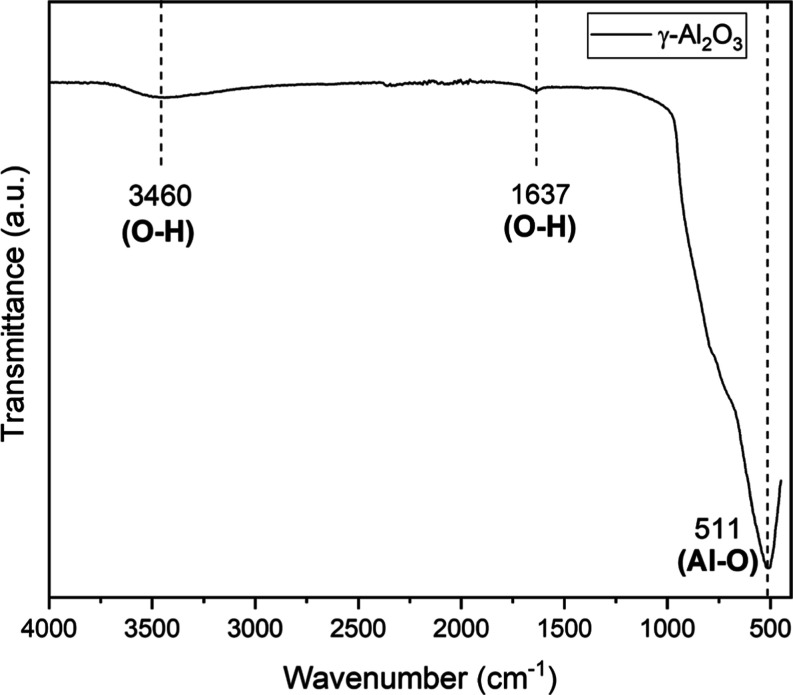
FTIR of γ-Al_2_O_3_.

### 3.4 Rheology of Nanofluids

The rheological behavior of the nanofluids is presented in Figure 8. The brine sample was included as a baseline fluid to allow direct comparison with the nanofluids. As shown in Figure 8a, all fluids exhibited a linear relationship between shear stress and shear rate, confirming Newtonian behavior. In Figure 8b, the viscosity remained nearly constant over the entire shear rate range for all samples, further supporting this classification.

**Figure 8:**
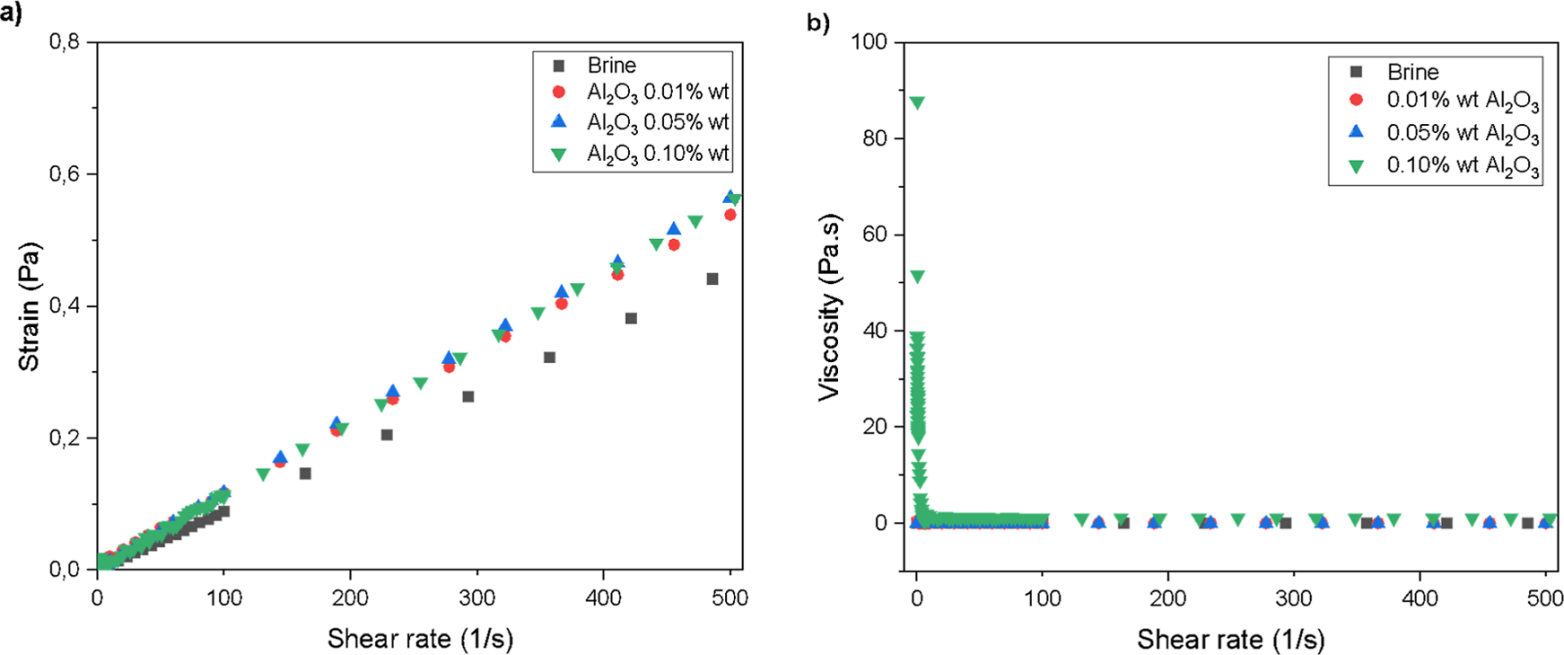
(a) Shear rate behavior with stress (b) shear rate behavior with viscosity of γ-Al_2_O_3_ nanofluids.

It is also evident that the addition of Al_2_O_3_ nanoparticles had a minimal effect on the steady-state viscosity at higher shear rates. However, at very low shear rates, a slight increase in initial viscosity was observed for the nanofluid containing 0.10 wt % Al_2_O_3_. This behavior may be attributed to enhanced hydrodynamic interactions among nanoparticles at higher concentrations, which can increase resistance to flow under low-shear conditions.^46^ Nonetheless, the viscosities of all nanofluids converge toward that of the base brine under operational shear rates, indicating their suitability for injection applications without significant additional pressure requirements.

### 3.5 Evaluation of Nanofluid Stability via UV–Vis Spectroscopy and Sedimentation Analysis

The UV–vis spectroscopic analysis shows that the nanofluid containing 0.01 wt % Al_2_O_3_ (Figure 9a) exhibits a well-defined absorbance peak around ∼202 nm, with only a slight decrease over time. This behavior suggests good dispersion and colloidal stability at this concentration, with minimal nanoparticle aggregation or sedimentation during the 2 h evaluation period.

**Figure 9:**
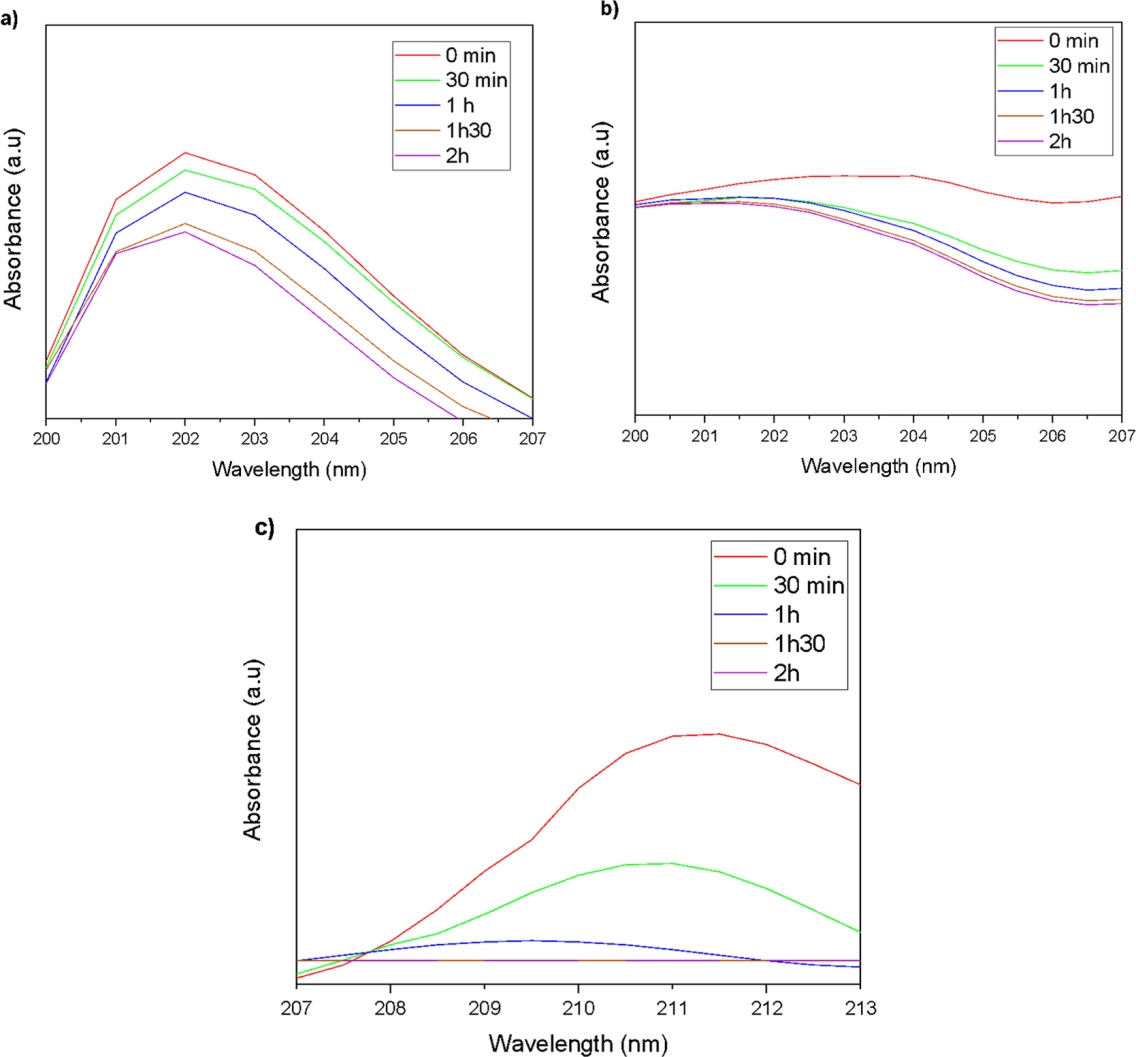
UV–vis absorbance spectra of γ-Al_2_O_3_ nanofluids at concentrations of (a) 0.01 wt %, (b) 0.05 wt %, and (c) 0.10 wt %, measured at 30 min intervals for a total of 2 h.

In contrast, the spectra for the 0.05% and 0.10% samples (Figure 9b,c) present smoother profiles and a more pronounced decline in absorbance over time. This effect is directly related to the reduction in the number of dispersed particles in the solution due to nanoparticle agglomeration and subsequent sedimentation.^47^

This trend is corroborated by the sedimentation test images (Figure 10). In the 0.01 wt % samples, no visible sediment is observed after 2 h of rest, indicating higher colloidal stability. However, in the 0.05% and especially the 0.10% samples, a clear sediment layer forms at the bottom of the vials after just 1 h, becoming more prominent by the 2 h mark. This behavior confirms that increasing the particle concentration leads to a greater tendency for aggregation and settling, which reduces the effectiveness of the nanofluid as a dispersed system.

**Figure 10:**
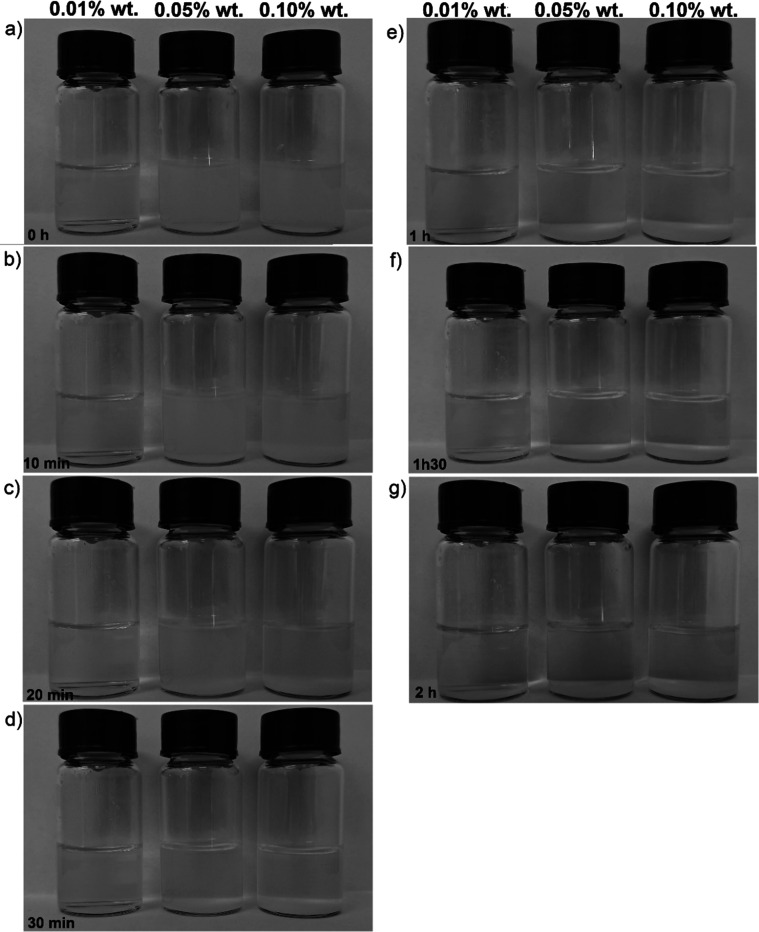
Visual sedimentation test of γ-Al_2_O_3_ nanofluids at concentrations of 0.01%, 0.05%, and 0.10% by weight, photographed at different times. From left to right, the vials correspond to increasing concentrations.

Therefore, the collected data indicates that although higher concentrations provide a greater amount of active material, colloidal stability becomes a limiting factor. The 0.01 wt % formulation demonstrated the best performance in terms of dispersion and temporal stability, making it more suitable for applications that require prolonged stability.

These results are consistent with the FESEM observations, which confirmed the agglomeration of alumina nanoparticles even when dispersed in nanofluids. This aggregation behavior explains the discrepancy between the particle size values estimated using the Scherrer equation and those observed in the FESEM images, where the latter appear significantly larger due to nanoparticle clustering.

### 3.6 Interfacial Tension

Figure 11 presents the interfacial tension (IFT) values between crude oil and nanofluids at different Al_2_O_3_ concentrations. The results, obtained in duplicate (*n* = 2), are reported as mean ± standard deviation.

**Figure 11:**
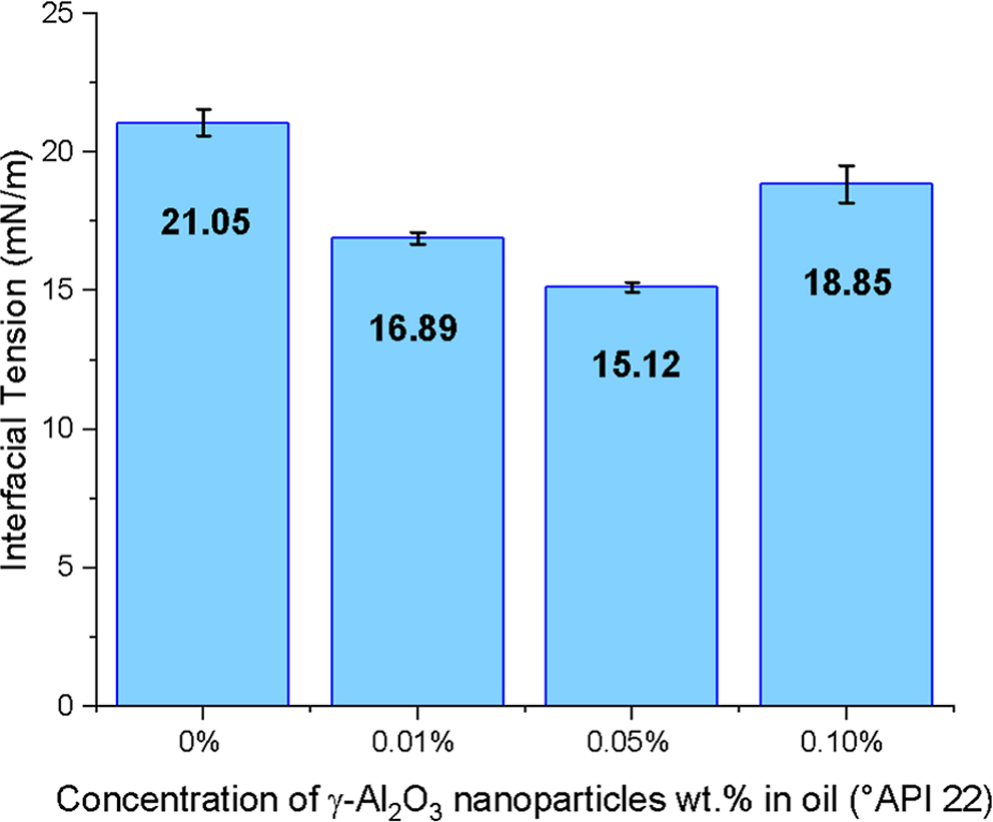
Interfacial tension test results.

The nanofluid containing 0.05 wt % Al_2_O_3_ exhibited the greatest reduction in IFT, reaching 15.12 ± 0.18 mN/m, compared to 21.05 ± 0.48 mN/m for the base brine solution. This indicates that 0.05 wt % was the most effective concentration for stabilizing the oil–water interface and reducing the adhesion forces between the phases. Nanoparticles play an important role in stabilizing the oil–fluid interface, preventing the precipitation and accumulation of asphaltenes, which are known to increase oil viscosity and reduce its mobility.^7^ The dispersion of asphaltenes in the oil prevents the formation of aggregates at the interface, favoring the reduction of IFT and, consequently, improving oil mobility.

At 0.01 wt %, the IFT was also significantly reduced (16.89 ± 0.22 mN/m), confirming the effectiveness of low concentrations. However, when the nanoparticle concentration was increased to 0.10 wt %, a slight increase in IFT was observed (18.85 ± 0.66 mN/m). This effect reduces the specific surface available to act on the oil–water interface, decreasing the interfacial and surface properties of the nanofluid.^48^

### 3.7 Wettability

Table 4 presents the contact angle values measured after 90 s for rock samples subjected to different saturation conditions: white (saturated only with oil), brine, and alumina nanofluids at concentrations of 0.01, 0.05, and 0.10 wt %. The results are shown for both nanofluid treatment durations (30 min and 24 h). A clear reduction in contact angle is observed in samples treated with nanofluids, indicating a wettability alteration toward more water-wet conditions, which becomes more pronounced with increased treatment time and concentration.

**Table 4:** Contact Angle Values Measured after 90 s for Rock Samples Treated with Alumina Nanofluids at Different Concentrations and Immersion Times (30 min and 24 h)

	treatment durations	
	30 min	24 h
saturation conditions	contact angle	contact angle
white	100.50 (±1.23)°	106.08 (±1.12)°
brine	94.00 (±2.28)°	109.39 (±2.80)°
NF 0.01%	90.90 (±0.81)°	64.00 (±0.67)°
NF 0.05%	87.2 (±2.60)°	0°
NF 0.10%	86.6 (±1.15)	0°

The surface treatment with brine alone did not result in significant wettability alteration, as evidenced by the increase in contact angle from 94.00° to 109.39° after 24 h. This suggests that brine, in isolation, is insufficient to induce a stable shift in the fluid–rock interaction. In contrast, the presence of Al_2_O_3_ nanoparticles significantly enhances wettability alteration, especially at longer treatment durations. For a 30 min exposure, nanofluids with 0.01 and 0.05 wt % reduce the contact angle to 90.90° and 87.2°, respectively, indicating a progressive transition toward a mixed-wet condition.^49,50^ However, after 24 h, a marked effect is observed: the 0.01 wt % nanofluid reduces the contact angle to 64.00°, while both 0.05 and 0.10 wt % reach a contact angle of 0°, indicating a complete wettability reversal.

These results suggest that not only nanoparticle concentration but also exposure time plays a critical role in wettability modification. The observed reductions in contact angle are comparable to or even surpass values reported in the literature for SiO_2_ nanofluids. For example, Joonaki and Ghanaatian (2014)^50^ demonstrated a reduction to 82° using a 1.5 g/L SiO_2_ nanofluid. Furthermore, the same study also reported improved wettability with the use of Al_2_O_3_ nanofluids, although with lower performance compared to SiO_2_ and to the results observed in the present work for alumina nanoparticles. This highlights the application potential of the nanoparticles synthesized via the hydrothermal method employed.

The wettability mechanism occurs when alumina nanoparticles interact with the sandstone surface by hydrogen bonding between hydroxyl groups present on the particles and functional groups on the rock substrate, promoting the replacement of previously adsorbed compounds.^30^ This surface modification gradually renders the rock more water-wettable, facilitating the mobilization of trapped oil and, consequently, enhancing the recovery efficiency,^48^ as illustrated in Figure 12.

**Figure 12:**
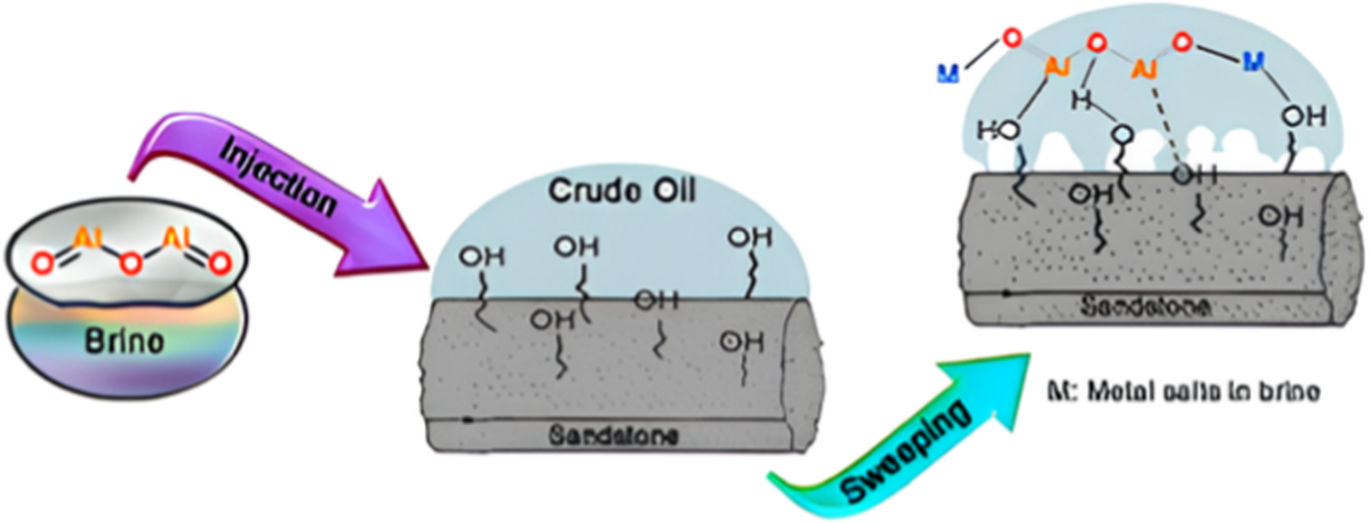
Mechanism for adsorption of alumina particles on sandstone core. *Reproduced with permission from Kiani, S.; Mansouri Zadeh, M.; Khodabakhshi, S.; Rashidi, A.; Moghadasi, J. Newly Prepared Nano Gamma Alumina and Its Application in Enhanced Oil Recovery: An Approach to Low-Salinity Waterflooding. Energy& Fuels 2016, 30 (5), 3791–3802.* 10.1021/acs.energyfuels.5b03008. *Copyright 2016 American Chemical Society.*

Studies indicate that adsorption can reduce porosity and alter the permeability of the rock, favoring the mobilization of oil during the injection process, since with the change in wettability the relative permeability of the oil tends to increase, while the relative permeability of the aqueous phase decreases, thus enhancing oil mobility in the porous network.^51–53^ In addition to the chemical effect of adsorption, nanoparticles can also influence wettability by electrostatic interactions, variations in rock surface roughness and formation of thin layers of nanofluids that modify the interaction between the oil phase and the porous matrix.^53,54^

### 3.8 Enhanced Recovery Tests

Five tests were performed using the brine and nanofluid at room temperature, with a constant flow rate of 0.25 cm^3^/min. Table 5 lists the test flow rate, permeability (*k*), porosity (Φ), and pore volume (PV) of the plugs used for each test and data on initial oil saturation (*S*_oi_), initial water saturation (*S*_wi_), and the oil recovery factor (% FR). A tendency for increased oil recovery was observed with the increase in the concentration of nanoparticles in the injected nanofluid. It is estimated that this occurs due to a greater surface area available to interact with the oil and reservoir rock, as shown by the IFT and wettability results present in this study and observed in other studies.^55,56^

**Table 5:** Experimental Results of Nanofluid Injection as an Enhanced Oil Recovery Method

	rock data			saturation data		
fluid type	*K* (mD)	Φ (%)	PV	*S*_oi_	*S*_wi_	FR (%)
brine	271.95	22.10	17.67	0.80	0.20	26.31
NF 0.01%	265.49	22.72	17.83	0.79	0.21	24.61
NF 0.05%	277.14	21.67	17.78	0.78	0.22	26.98
NF 0.10%	271.49	21.46	17.63	0.77	0.23	29.71
brine + NF 0.10%	272.6	21.45	17.60	0.77	0.23	28.96

Figure 13 presents the relationship between accumulated oil recovery and the injected pore volume (PV) for the different fluids tested.

**Figure 13:**
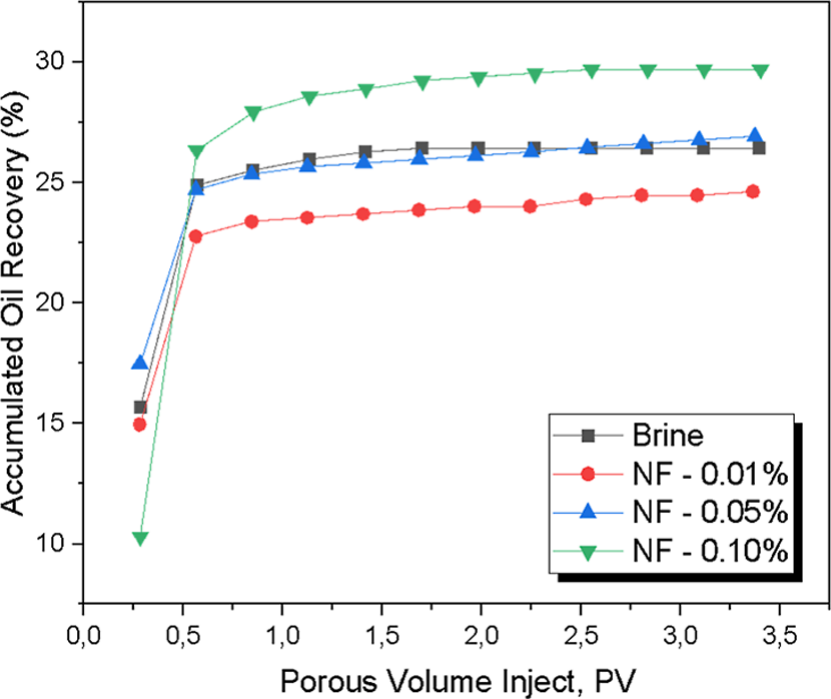
Ratio between accumulated oil recovery and injected pore volume of different fluids.

It is observed that brine resulted in a recovery factor of approximately 26%, stabilizing after the injection of 1 PV. The nanofluid with a 0.01% concentration exhibited slightly lower performance compared to brine, indicating that the low nanoparticle content was insufficient to induce significant changes in oil mobility. This behavior may be associated with the limited dispersion and adsorption of nanoparticles within the rock matrix. At such low concentrations, the number of active nanoparticles may be too limited to promote mechanisms typically responsible for enhanced recovery, such as wettability alteration and interfacial tension reduction.^56^ In addition, weak adsorption or partial retention of nanoparticles in the porous medium may hinder oil displacement, leading to a performance similar to, or slightly below, that of the base fluid (brine). Therefore, the observed result reinforces the concentration-dependent nature of nanofluid performance in EOR applications.^57–59^

When the concentration increased to 0.05%, a slight improvement in the recovery factor was observed compared to brine, possibly due to enhanced nanoparticle adsorption in the sandstone pores. This adsorption may contribute to changes in rock wettability and reduced oil retention within the porous medium.^15,60^

The 0.10% nanofluid showed the best performance among the tested fluids, achieving an oil recovery greater than 29%, and compares favorably with values reported in the literature for other metal oxide nanofluids.^61^ For instance, TiO_2_ nanoparticles at 0.01 wt % achieved recovery factors ranging from 14% to 31% depending on core permeability.^62,63^ CuO nanofluids at 0.2 wt % reached an average recovery of 11.65% in sandstone and 6.92% in carbonate rocks.^64^ Similarly, SnO_2_ nanoparticles applied in carbonate formations yielded a maximum of 24% recovery.^65^ These comparisons indicate that the γ-Al_2_O_3_ system investigated in this study performs at the upper end of the range reported for metal oxide-based nanofluids under similar core-flooding conditions, reinforcing its potential for enhanced oil recovery.

Although the percentage increase from 26% to 29% may appear modest, it represents a potentially relevant improvement in oil volume when extrapolated to reservoir scale, potentially resulting in relevant economic benefits. This improvement is likely related to the reduction in interfacial tension and the alteration of wettability, as previously observed in fluid characterizations, which promote enhanced oil mobility within the porous rock.

However, these results also highlight a critical trade-off: while increasing nanoparticle concentration tends to improve oil recovery, it also raises the risk of particle agglomeration, which compromises nanofluid stability, reduces injectivity, and may lead to pore blockage. In addition to agglomeration, potential nanoparticle retention within the porous medium should also be considered. Although no pressure buildup or flow interruption was observed during these experiments, which were conducted at low injection rates and with stable nanofluid formulations, the possibility of pore-scale retention or plugging cannot be fully excluded. Further studies involving effluent analysis and postflood core characterization are recommended to quantify retention effects and assess potential formation damage under reservoir conditions. The moderate increase in recovery may also be associated with the interaction between brine salinity and nanofluid stability. Studies indicate that high salinity levels favor nanoparticle agglomeration, which compromises their ability to alter rock wettability.^29,66,67^

Therefore, concentrations above 0.10% were not evaluated due to the risk of particle agglomeration, which could undermine fluid stability and increase production costs. Future studies may explore the use of dispersing or stabilizing agents to enable the application of higher nanoparticle concentrations without compromising performance.^66,68,69^ It is also noticeable that, across all curves, oil recovery increases significantly up to approximately 0.4 PV injected. Beyond this point, the increase in the recovery factor becomes more gradual until it stabilizes, indicating that additional oil production becomes negligible as injection continues.

To investigate the practical applicability of the most efficient nanofluid (NF-0.10%) as a secondary recovery method, a new injection was carried out following conventional brine flooding. Figure 14 shows the comparison between brine alone, isolated NF-0.10%, and the sequential combination of brine followed by NF-0.10%.

**Figure 14:**
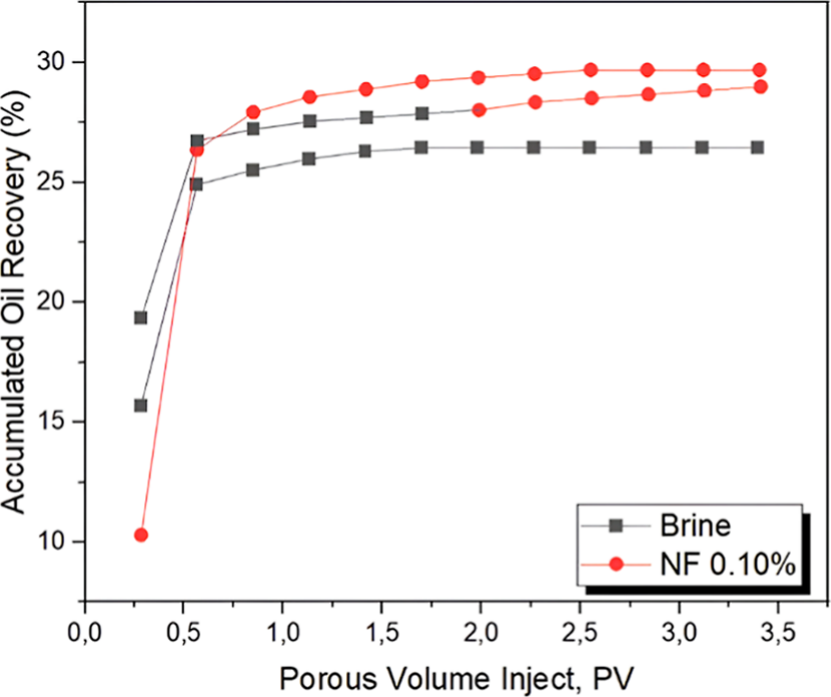
Comparison of the recovery factor between brine, the nanofluid at the highest concentration, and their combination.

The analysis reveals that the direct injection of the nanofluid achieved the highest oil recovery (approximately 30%). The brine + NF-0.10% combination showed slightly lower performance but still outperformed brine alone. This strategy can be considered economically viable, as it achieves results comparable to those of isolated nanofluid injection while using only half the weight of nanoparticles, thereby reducing process costs.

The results reinforce that the addition of nanoparticles to the injected fluid enhances oil recovery, provided that the formulation and concentration are properly optimized. Very low concentrations may not produce significant effects, while suitable concentrations demonstrate a positive impact on oil mobility. Meanwhile, the interaction between nanoparticles and brine requires further investigation, as it may directly influence the efficiency of the recovery process.

## 4 Conclusions

The results presented herein confirm the production of γ-Al_2_O_3_ nanoparticles with high surface area, achieved at low temperature (175 °C) and within a short processing time (1 h). XRD analysis, supported by Rietveld refinement, revealed the transformation of boehmite into γ-Al_2_O_3_ upon thermal treatment, resulting in a pure cubic phase with no secondary phases detected, demonstrating good structural quality. Morphological analysis by FEG-SEM confirmed the formation of nanoplate structures, although particle agglomeration was observed. This agglomeration explains the discrepancy between the crystallite size estimated by the Scherrer equation (∼6 nm) and the larger structures observed by FEG-SEM, indicating that the nanoplates consist of polycrystalline aggregates.

BET analysis confirmed high specific surface area of 101.91 m^2^/g, a key feature for applications in oil recovery. Rheological measurements indicated Newtonian behavior and low viscosity for all nanofluid formulations, which enhances efficient injection and flow through porous media in enhanced oil recovery (EOR) operations.

UV–vis absorbance and sedimentation tests showed that colloidal stability decreases as the concentration of nanoparticles increases. While the 0.01 wt % nanofluid remained stable with minimal sedimentation over 2 h, higher concentrations (0.05% and 0.10%) exhibited significant aggregation and sedimentation. These findings suggest that particle concentration plays a crucial role in nanofluid stability and, consequently, in its performance.

Interfacial tension measurements demonstrated that γ-Al_2_O_3_ nanofluids can effectively reduce the oil–water interfacial tension, potentially enhancing oil mobility and preventing asphaltene precipitation. The wettability changes confirmed the effectiveness of γ-Al_2_O_3_ nanofluids in enhancing water-wet conditions, especially with increased concentration and prolonged treatment time. After 30 min, nanofluids with 0.01 and 0.05 wt % reduced the contact angle to 90.90° and 87.2°, respectively, indicating a shift toward mixed-wet behavior. After 24 h, the 0.01 wt % nanofluid further reduced the angle to 64.00°, while 0.05 and 0.10 wt % reached 0°, demonstrating complete wettability reversal and strong potential for improved oil recovery.

In conclusion, nanofluids containing hydrothermally synthesized γ-Al_2_O_3_ show strong potential for application in EOR processes. However, the effectiveness of these fluids is highly dependent on colloidal stability. Although higher concentrations offer enhanced recovery, they also promote aggregation and sedimentation, which can hinder fluid performance. Therefore, incorporating nanoparticle stabilizers, such as surfactants or dispersing agents can potentially maintain dispersion stability and optimize oil recovery efficiency.
